# *In vitro* and *Quantitative and Structure Activity Relationship* (QSAR) evaluation of the antifungal activity of terpenoid constituents of essential oils against *Alternaria alternata* and *Fusarium oxysporum*

**DOI:** 10.7705/biomedica.6883

**Published:** 2023-08-31

**Authors:** Sergio Andrade-Ochoa, Daniela Sánchez-Aldana, Luz María Rodríguez-Valdez, Guadalupe Virginia Nevárez-Moorillón

**Affiliations:** 1 Facultad de Ciencias Químicas, Universidad Autónoma de Chihuahua, Chihuahua, México Universidad Autónoma de Chihuahua Facultad de Ciencias Químicas Universidad Autónoma de Chihuahua Chihuahua Mexico; 2 Facultad de Medicina y Ciencias Biomédicas, Universidad Autónoma de Chihuahua, Chihuahua, México Universidad Autónoma de Chihuahua Facultad de Medicina y Ciencias Biomédicas Universidad Autónoma de Chihuahua Chihuahua Mexico

**Keywords:** Fusarium, Alternaría, oils, volatile, quantitative structureactivity relationship, Fusarium, Alternaría, aceites volátiles, relación estructura-actividad cuantitativa

## Abstract

**Introducción.:**

Los géneros *Alternaria* y *Fusarium* contienen especies patógenas para los humanos y los cultivos. Para su control, se han utilizado diversos antifúngicos. Sin embargo, su uso desmedido ha contribuido al desarrollo de agentes patógenos resistentes. Una alternativa para buscar y desarrollar nuevos agentes antimicóticos son los aceites esenciales y sus componentes principales, los cuales poseen diversas actividades biológicas de interés para la medicina y en la preservación de alimentos.

**Objetivo.:**

Evaluar *in vitro* e *in silico* las actividades antifúngicas de terpenoides contra *Alternaria alternata* y *Fusarium oxysporum.*

**Materiales y métodos.:**

Se evaluaron *in vitro* las concentraciones inhibitorias mínimas y las concentraciones fungicidas mínimas de 27 constituyentes de aceites esenciales contra *A. alternata* y *F. oxysporum.* Además, mediante algoritmos genéticos, se crearon modelos cuantitativos de la relación estructura-actividad para determinar las propiedades estructurales y fisicoquímicas relacionadas con la actividad antifúngica.

**Resultados.:**

Los compuestos evaluados mostraron ser antifúngicos activos. El timol fue el compuesto con mayor actividad, con un valor de concentración inhibitoria mínima de 91.6 ± 28.8 pg/ml, tanto para *Alternarla alternata* como para *Fusarium oxysporum.* Los modelos cuantitativos de la relación estructura-actividad incluyeron la avidez por los lípidos y los fenoles como los principales grupos funcionales que contribuyen en la actividad antifúngica.

**Conclusión.:**

Los terpenoides poseen actividades antifúngicas relevantes para ser incorporados en el estudio de la química medicinal. La inclusión de pruebas *in silico* a la evaluación *in vitro* es una herramienta útil para la búsqueda y el diseño racional de derivados terpénicos como posibles agentes antifúngicos.

## Resumen

**Introduction.:**

Fungal *genera Alternaría* and *Fusarium* include human and plant pathogenic species. Several antifungals have been used for their control, hut excessive use has contributed to resistance development in pathogens. An alternative to searching for and developing new antifungal agents is using essential oils and their main components, which have biological activities of interest in medicine and food production.

**Objective.:**

To evaluate *in vitro* and *in silico* the antifungal activities of terpenoids against *Alternaria alternata* and *Fusarium oxysporum.*

**Materials and methods.:**

The minimum inhibitory concentration and minimum fungicidal concentration values of 27 constituents of essential oils used against *Alternaria alternata* and *Fusarium oxysporum* were evaluated *in vitro.* In addition, using genetic algorithms, quantitative models of the structure-activity relationship were used to identify the structural and physicochemical properties related to antifungal activity.

**Results.:**

The evaluated compounds proved to be effective antifungals. Thymol was the most active with a minimum inhibitory concentration of 91.6 ± 28.8 pg/ml for *A. alternata* and *F. oxysporum.* Quantitative structure-activity relationship models revealed the octanol-water cleavage ratio as the molecular property, and the phenols as the main functional group contributing to antifungal activity.

**Conclusion.:**

Terpenoids exhibit relevant antifungal activities that should be incorporated into the study of medicinal chemistry. Inclusion of *in silico* assays in the *in vitro* evaluation is a valuable tool in the search for and rational design of terpene derivatives as new potential antifungal agents.

*Alternaría* and *Fusarium* genera include human pathogenic species and pathogens causing wilting or root and crown rot in economically important crops ^1^^,^^2^. *Alternaría alternata* is an airborne fungal species continually reported in nasal secretions and with vigorous immunological activity in nasal epithelial cells, where it plays an essential role in the pathogenesis of chronic rhinosinusitis ^3^. In addition, *A. alternata* is recognized as an opportunistic mold responsible for skin lesions in patients under corticosteroid treatment, suffering from Cushing's syndrome, undergoing kidney, liver, or bone marrow transplants, and presenting solid and hematological neoplasms, aplastic anemia, or AIDS. Furthermore, several mycotoxins and secondary metabolites produced by *A. alternata* have been isolated from a wide range of harvested food products ^4^.

On the other hand, *Fusarium oxysporum,* a filamentous, hyaline, septate fungus belonging to the group of hyalohyphomycosis agents, is recognized as the cause of localized infections, such as keratitis and disseminated infections, with multisystemic involvement in immunocompromised patients ^5^ . Similarly, these pathogens can affect ornamental and many garden crops ^6^ and are responsible for pre- and post-harvest diseases ^7^, generating significant economic losses ^8^.

Antifungals have been used in medicine and agriculture to control several pathogenic fungi ^9^. The widespread use of antifungals has inhibited the outbreak of fungal diseases but, at the same time, it has contributed to the development of resistant pathogens ^10^, as in the case of *A. alternata* or *Alternata solani,* which exhibits resistance against succinate dehydrogenase inhibitors ^11^^,^^12^. Strains of *A. alternata* resistant to fluconazole and 5fluorocytosine have also been reported ^13^. *Fusarium* species are intrinsically resistant to azole antifungals ^14^, but some studies described resistance to echinocandins and polyenes ^15^.

Some essential oils display relevant antifungal activities ^16^^,^^17^. There are reports of essential oils exhibiting antifungal activity against *Candida* spp. ^18^, *Aspergillus* spp. ^19^, *Alternaria* spp. ^20^, and *Fusarium* spp. ^21^^,^^22^, among other pathogenic fungi ^23^^-^^25^. Essential oils have broad applications in folk medicine and food preservation ^26^. Volatile compounds in essential oils, such as terpenoids, and aliphatic and aromatic compounds, constitute a rich source of potential agents against pathogenic microorganisms.

Currently, it is possible to use computational tools to analyze the biological activity of compounds with pharmacological potential to accelerate new antimicrobial agents' development. Computer-aided drug design is a valuable technique that saves time and resources in the design process of new drugs ^27^. Computational models can identify the most promising compounds for further development, reducing the testing and experimentation required to obtain an effective drug ^28^.

Among the computational tools for drug development are quantitative and structureactivity relationship (QSAR) models. This technique is based on the idea of a quantitative relationship between the chemical structure of a compound and its biological activity, which implies that changes in a compound chemical structure can affect its biological activity in a predictable way ^29^. In addition, QSAR studies can help identify the underlying molecular mechanisms affecting a compound biological activity, providing a better understanding of how compounds interact and how they can be modified to improve their efficacy ^30^.

In light of the above, this study aimed to evaluate the antifungal activity of terpenoids against *A. alternata* and *F. oxysporum* using *in vitro* bioassays. In addition, QSAR studies were conducted between the physicochemical properties of the compounds and their antifungal effects.

## Materials and methods

### In vitro evaluations

**Figure 1:**
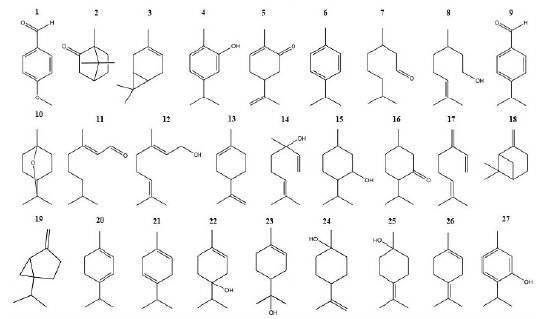
Chemical structure of evaluated terpenoids. ^1^ p-Anisaldehyde, ^2^ Canphor, ^3^ 3 Carene, ^4^ Carvacrol, ^5^ Carvone, ^6^ β -Cymene, ^7^ Citronellal, ^8^ β-Citronellol, ^9^ Cuminaldehyde, ^10^ Eucalyptol, ^11^ Geranial, ^12^ Geraniol, ^13^ Limonene, ^14^ Linalool, ^15^ Menthol, ^16^ Menthone, ^17^ β -Myrcene, ^18^ β -Pinene, ^19^ Sabinene, ^20^ α-Terpinene, ^21^ y-Terpinene, ^22^ 4-Terpineol, ^23^ α -Terpineol, ^24^ β -Terpineol, ^25^ y-Terpineol, ^26^ α Terpinolene, ^27^ Thymol.

The antifungal activity of 27 compounds was evaluated against environmental strains of *A. alternata* and *F. oxysporum* isolated in the Microbiology III Laboratory of the *Facultad de Ciencias Químicas* of the *Universidad Autónoma de Chihuahua.* The compounds evaluated were obtained from Sigma-Aldrich (México City, México) and are presented in figure 1.

Minimum inhibitory concentration values were determined following the methodology described by Rasooli and Mirmostafa ^31^, and Rasooli *et al.*^32^, with certain modifications.

Fungal strains were plated in Sabouraud medium and incubated for two weeks at 37 °C. After incubation, spores were collected by adding 20 ml of phosphate buffer to the plate with 0.5% Tween 80. A spore count was conducted using a Neubauer chamber to adjust the number of spores to the required concentration.

To determine minimum inhibitory concentration values using the microdilution on microplate technique, we added to the microplate wells 100 µl of Sabouraud broth containing different concentrations of the selected compounds (1,250, 1,100, 1,000, 750, 500, 250, 100, 75, 50, and 25 µg/ ml). Subsequently, 5 x 10^5^ spores, suspended in 100 µl of Sabouraud broth, were added to the wells to obtain a final volume of 200 µl in each. The plates were incubated at 28 °C for 48 hours. Minimum inhibitory concentration corresponded to the highest without visible mold growth observed. Peripheral wells were filled with 200 µl sterile distilled water to minimize medium dehydration. The wells of column 11 were filled with 200 µl of medium and 500,000 spores, serving as positive controls for the experiment.

Minimum fungicidal concentration values were calculated by drawing 50 µl from the microwells without fungal growth and pouring them into a Petri dish with Sabouraud medium. We calculated the minimum fungicidal concentration as the one with no fungal growth. Each test was performed in triplicate.

### In silico evaluations

The computational study was carried out with the methodology previously described ^33^^,^^34^. Structural, constitutional, physicochemical, and topological descriptors were generated using Dragon 5.4 software ^35^, and Koopman's theorem ^36^ was applied to calculate the chemical reactivity descriptors. All molecules were analyzed in the aqueous phase. The polarizable continuum model was used to model the solvent effects ^37^.

QSAR studies were carried out using all biological activities obtained from the *in vitro* tests and the calculated theoretical descriptors. The analysis was conducted using genetic algorithms with MobyDigs software ^38^. The quality of the model was considered statistically satisfactory based on the determination coefficient (R^2^), leave-one-out cross-validated explained variance (Q^2^), standard deviation (SD), and the ANOVA (F) of the model.

## Results

### Fungicidal activity

We evaluated the inhibitory and fungicidal activity of 27 terpenoids and structurally similar molecules that are major components of essential oils. The results showed that thymol exhibited the most relevant inhibitory activity, with a minimum inhibitory concentration of 91.6 ± 28.8 µg/ml for both *A. alternata* and *F. oxysporum* (table 1). Thymol was also the most relevant for its fungicidal effect, displaying slight differences in activity, with minimum fungicidal concentration values of 150 ± 28.8 µg/ml for *A. alternata* and 316.6 ± 57.7 µg/ml for *F. oxysporum* (table 2). Carvacrol, an isomer of thymol, was the compound with the second most relevant inhibitory and fungicidal activity, with a minimum inhibitory concentration of 200 ± 86.6 and 283 ± 86.6 µg/ml for *A. alternata* and *F. oxysporum,* respectively.

**Table 1:** Fungicidal activity of terpenoids and related compounds against *Alternarla alternata.* Minimum inhibitory concentration (MIC) and minimum fungicidal concentration (MFC) are expressed in µg/ml.

*Altemarìa alternata*				
No.	Compounds	MIC	MFC	Grouping
T27	Thymol	91.6 ± 14.4	150 ± 28.8	I
T4	Carvacrol	200 ± 86.6	550 ± 86.6	HI
T1	Anisaldehyde	416.6 ± 57.7	683.3 ± 28.8	GH
T5	Carvone	416.6 ± 57.7	783.3 ± 57.7	GH
T17	β-Myrcene	466.6 ± 0.0	500 ± 0	FG
T22	4-Terpineol	466.7 ± 57.7	1000 ± 0	FG
T3	3-Carene	483.3 ± 28.8	966.6 ± 28.8	FG
T9	Cuminaldehyde	483.3 ± 28.8	966.6 ± 28.8	FG
T26	α-Terpinolene	483.3 ± 57.7	1,000 ± 0	FG
T23	α-Terpineol	483.3 ± 0	1,000 ± 0	FG
T2	Camphor	500± 0	950 ± 0	EFG
T12	Geraniol	516.6 ± 115.4	816.6 ± 115.4	EFG
T20	α-Terpinene	550 ± 0	1,000 ± 0	EFG
T24	β-Terpineol	550 ± 57.7	1,000 ± 0	EFG
T25	γ-Terpineol	633.3 ± 28.8	1,000 ± 0	DEFG
T15	Menthol	666.7 ± 28.8	983.3 ± 28.8	CDEFG
T21	γ-Terpinene	683.3 ± 28.8	1000 ± 0	CDEF
T11	Geranial	683.3 ± 28.8	966.6 ± 28.8	CDEF
T10	Eucalyptol	716.6 ± 28.8	983.3 ± 28.8	BCDEF
T8	β-Citronellol	750 ± 28.7	966.6 ± 28.8	BCDE
T14	Linalool	750 ± 28.8	966.6 ± 28.8	BCDE
T13	Limonene	816.6 ± 57.7	1,033.3 ± 57.7	ABCD
T19	Sabinene	883.3 ± 0	1000 ± 0	ABCD
T16	Menthone	883.3 ± 57.7	1,033.3 ± 57.7	ABCD
T7	Citronellal	900 ± 0	1,250 ± 0	ABC
T18	β-Pinene	966.8 ± 0	1,350 ± 0	AB
T6	p-Cymene	1,033.33 ± 0	1,250 ± 0	A

Grouping information was performed using the Tukey method and 95% confidence using the MIC values. Compounds that do not share a letter are significantly different.

**Table 2:** Fungicidal activity of terpenoids and related compounds against *Fusarlum oxysporum.* Minimum inhibitory concentration (MIC) and minimum fungicidal concentration (MFC) are expressed in µg/ml.

*Fusarium oxyporum*				
No.	Compounds	MIC	MFC	Grouping
T27	Thymol	91.6 ± 28.8	316.6 ± 57.7	I
T4	Carvacrol	283.3 ± 86.6	400 ± 86.6	HI
T12	Geraniol	483.3 ± 57.7	783.3 ± 57.7	GH
T23	α-Terpineol	500 ± 144.3	916.7 ± 144.3	GH
T24	β-Terpineol	583.3 ± 0	1,000 ± 0	FG
T10	Eucalyptol	633.3 ± 57.7	1,033.3 ± 57.7	EFG
T20	α-Terpinene	650 ± 57.7	816.6 ± 57.7	EFG
T2	Camphor	650 ± 86.6	1,200 ± 86.6	EFG
T3	3-Carene	650 ± 86.6	1,200 ± 86.6	EFG
T17	β-Myrcene	666.6 ± 57.7	883.3 ± 57.7	EFG
T22	4-Terpineol	666.6 ± 57.7	1,033.3 ± 57.7	EFG
T11	Geranial	700 ± 0	1,000 ± 0	DEFG
T13	Limonene	716.6 ± 125.8	1,083.3 ± 125.8	CDEFG
T26	α-Terpinolene	750 ± 0	750 ± 0	BCDEFG
T21	γ-Terpinene	783.3 ± 0	1,000 ± 0	BCDEF
T15	Menthol	783.3 ± 57.7	1,066.6± 57.7	BCDEF
T25	γ-Terpineol	783.3 ± 0	1,000 ± 0	BCDEF
T14	Linalool	816.6 ± 57.7	1,033.3 ± 57.7	BCDEF
T9	Cuminaldehyde	833.3 ± 202.7	1,283.3 ± 125.8	BCDEF
T18	β-Pinene	866.6 ± 86.6	1,166.6 ± 125.8	BCDE
T16	Menthone	900 ± 186.6	1,083.3 ± 86.6	BCDE
T1	Anisaldehyde	950 ± 100	1,100 ± 100	ABCD
T19	Sabinene	950 ± 0	1,250 ± 0	ABCD
T5	Carvone	983.3 ± 0	1,250 ± 0	ABC
T6	p-Cymene	983.3 ± 0	1,250 ± 0	ABC
T8	β-Citronellol	1,000 ± 0	1,250 ± 0	AB
T7	Citronellal	1,200 ± 144.3	1,416.6 ± 125.8	A

Grouping information was performed using the Tukey method and 95% confidence using the MIC values. Compounds that do not share a letter are significantly different.

Most of the evaluated compounds presented minimum inhibitory concentrations between 500 and 900 µg/ml, while the minimum fungicidal concentrations ranged from 750 to over 1,000 µg/ml. Compounds displaying less activity were p-cymene for *Alternaria* spp. and citronellal for *Fusarium* spp. Tables 1 and 2 present the compounds' minimum inhibitory concentration and minimum fungicidal concentration values. The compounds are listed from the highest to the lowest antifungal activity to facilitate the analysis.

**Table 3:** Summary of the statistics of quantitative structure-property-fungicidal activity relationship models against *Alternarla alternata*

Statistical parameter		QSAR			QPAR	
	Model 1	Model 2	Model 3	Model 4	Model 5	Model 6
n	27	27	27	27	27	27
Q^2^	67.8	66.7	59.21	73.21	70.2	68.9
R^2^	82.65	82.52	82.31	87.78	86.98	85.87
F	19	18.9	18.6	23.8	21	20.8
SD	0.108	0.108	0.108	0.78	0.85	0.94
Descriptors	Contributions					
	Model 1	Model 2	Model 3	Model 4	Model 5 Model 6	
nCt	0.0824	0.0687	WC	WC	WC	WC
nRCO	-0.1076	WC	WC	WC	WC	WC
nArOH	0.6793	0.5963	0.6687	WC	WC	WC
nOHt	-0.1487	-0.0904	-0.1304	WC	WC	WC
nArCHO	WC	0.1723	0.2026	WC	WC	WC
nCconj	WC	WC	0.08572	WC	WC	WC
UNIP	WC	WC	WC	0.0435	0.5847	0.0324
Ui	WC	WC	WC	0.293	0.2812	0.2627
Hy	WC	WC	WC	-0.4902	-0.5682	-0.4753
AMR	WC	WC	WC	-0.0275	WC	WC
J	WC	WC	WC	WC	-0.3045	WC
Qtot	WC	WC	WC	WC	WC	-0.1052
Intercept	2.7269	2.7483	2.9291	3.2548	2.3392	2.4404

n: Number of systems evaluated; Q^2^: The square of the coefficient of cross-validation; R^2^: The square of the correlation coefficient; F: Fisher statistic; SD: Standard deviation; ^WC^: Without contribution; nCt: Number of total tertiary C (sp^3^); nRCO: Number of ketones (aliphatic); nArOH: Number of aromatic hydroxyls; nOHt: Number of tertiary hydroxyl groups; nArCHO: Number of aromatic aldehydes; nCconj: Number of non-aromatic conjugated C (sp^2^); UNIP: Unipolarity; Ui: Unsaturation index; Hy: Hhydrophilic factor; AMR: Ghose-Crippen molar refractivity; J: Balaban-like index; Qtot: Total absolute charge (electronic charge index-ECI)

### Quantitative structure-activity relationship

Through a structure-property relationship approach, the present work generated mathematical models to determine the structure-property characteristics contributing to the inhibitory activity of terpenoids against *A. alternata* and *F. oxysporum.* For this, multiple linear regressions were generated using genetic algorithms with only structural descriptors (QSAR models) and incorporating topological, physicochemical, and chemical reactivity properties (QPAR models).

**Figure 2:**
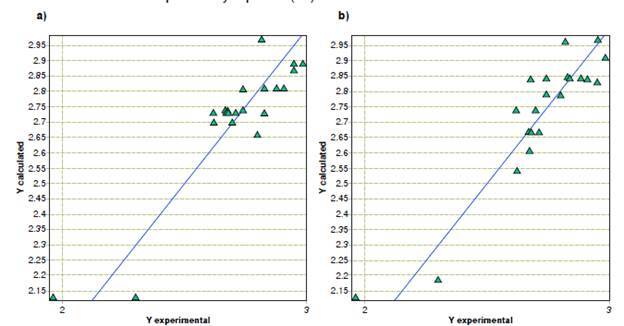
Predicted *versus* experimental activity against *Alternarla alternata* from (a) quantitative structure-activity relationship, and (b) quantitative property-activity relationship models.

In the case of *A. alternata,* linear multiple regression models found a direct relationship between minimum inhibitory concentration values and phenolic groups, benzaldehydes, and the number of tertiary carbons, while their relationships with the number of ketonic groups and tertiary alcohols were indirectly proportional. Regarding the molecular properties, we observed that the topological descriptors unipolarity and the unsaturation index contributed to the inhibitory activity. In contrast, physicochemical properties, such as the hydrophilic factor, Ghose-Crippen molar refractivity, and total absolute charge, exhibited an inverse relationship with activity. Table 3 shows the contribution of each descriptor value. The quality of the model is indicated by its statistical values. A plot of the predicted *versus* experimental activity for molecules, using a training set for models of *A. alternata,* is shown in figure 2.

**Figure 3:**
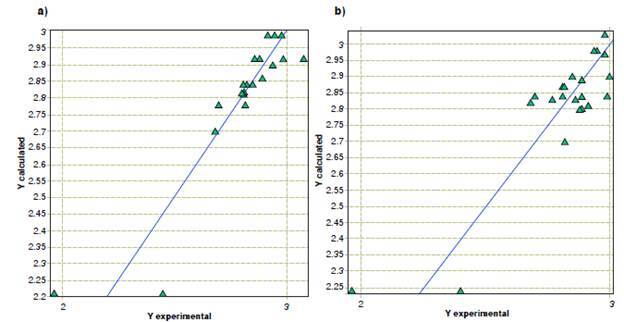
Predicted versus experimental activity against *Fusarium oxysporum* from (a) quantitative structure-activity relationship, and (b) quantitative property-activity relationship models

Models of the inhibitory activity of terpenic compounds and derivatives against *F. oxysporum* yielded similarities to the activity against *A. alternata.* First, the structural components that contribute the most to the inhibitory activity are the phenolic groups, but in this case, the number of conjugated non-aromatic carbons is more relevant than the benzaldehydes. Regarding molecular properties, the topological descriptors unipolarity and unsaturation index also contribute to the inhibitory activity. However, the models for *F. oxysporum* showed that the Ghose-Crippen octanol-water partition coefficient is the physicochemical property that contributes the most, while the hydrophilic factor and the Ghose-Crippen molar refractivity descriptors continue to have an indirect relationship with the minimum inhibitory concentration. A plot of the predicted *versus* experimental activity for molecules, using a training set for F. *oxysporum* models, is shown in figure 3. The statistics of the models generated by the analysis of the genetic algorithms are shown in table 4.

**Table 4:** Summary of the statistics of quantitative structure-property-larvicidal activity relationship models against *Fusarium oxysporum.*

Statistical parameter		QSAR			QPAR	
	Model 1	Model 2	Model 3	Model 4	Model 5	Model 6
*n*	27	27	27	27	27	27
*Q* ^*2*^	68.48	68.48	61.74	64.66	64.25	60.9
*R* ^*2*^	83.6	83.6	82.55	83.54	82.2	80.94
*F*	20.4	20.4	18.9	18.9	17.6	12.7
*SD*	0.105	0.105	0.109	0.123	0.123	0.126
Descriptors	Contributions					
	Model 1	Model 2	Model 3	Model 4	Model 5	Model 6
nCt	0.09185	WC	0.0525	WC	WC	WC
nRCO	WC	-0.1018	WC	WC	WC	WC
nArOH	0.7896	0.7896	0.668	WC	WC	WC
nOHt	-0.13508	-0.13508	WC	WC	WC	WC
nArCHO	WC	WC	0.149	WC	WC	WC
nCconj	0.0794	0.0794	0.0549	WC	WC	WC
UNIP	WC	WC	WC	0.0535	0.5652	WC
Ui	WC	WC	WC	0.1909	0.2538	0.2234
AlogP	WC	WC	WC	0.2492	WC	0.2591
Hy	WC	WC	WC	WC	-0.4842	WC
AMR	WC	WC	WC	-0.0394	-0.0437	-0.0484
Pol	WC	WC	WC	WC	WC	-0.0453
Intercept	2.9946	2.9946	3.0307	4.2175	3.7956	4.0975

n: Number of systems evaluated; Q^2^: The square of the coefficient of cross-validation; R^2^: The square of the correlation coefficient; F: Fisher statistic; SD: Standard deviation; ^WC^: Without contribution; nCt: Number of total tertiary C (sp^3^); nRCO: Number of ketones (aliphatic); nArOH: Number of aromatic hydroxyls; nOHt: Number of tertiary hydroxyl groups; nArCHO: Number of aromatic aldehydes; nCconj: Number of non-aromatic conjugated C (sp^2^); UNIP: Unipolarity; Ui: Unsaturation index; AlogP: Ghose-Crippen octanol-water partition coefficient; Hy: Hydrophilic factor; AMR: Ghose-Crippen molar refractivity; Pol: Number of polarity

A complete theoretical characterization of the evaluated molecules, with the precise value of each descriptor incorporated into the QSAR models, has been previously reported ^33^.

## Discussion

In the second half of the 20^th^. century, the prevalence of fungal infections steadily increased due to a higher number of immunosuppressed patients and the generalization of diagnostic practices and aggressive therapies ^39^. Although information on diseases caused by filamentous fungi is limited, reports of infections by fungi belonging to the *genus Aspergillus* are more frequent ^40^. However, the incidence of infections by other *genera,* including *Fusarium* and *Alternaría*^41^, has increased in recent years.

New pathogenic fungal species have recently been described, and invasive fungal infections with *in vitro* resistance to antifungals, that were previously nonpathogenic or only caused superficial infections, are becoming more frequent ^42^.

In the search for new agents with antimicrobial activity, essential oils provide a potential alternative for controlling pathogenic microorganisms. Essential oils and their components have been extensively studied as antibacterial and antifungal agents in the food safety context and are being widely explored as insecticidal agents ^43^. However, it was not until the last decade that researchers studied their antimicrobial properties from a medicinal chemistry point of view by integrating them into studies of rational design, elucidating their mechanisms of action, and evaluating their antimicrobial activities using *in vitro, in silico,* and *in vivo* models.

Essential oils may represent one of the most promising natural products for fungal inhibition ^44^. Essential oils obtained from different plants or herbs have been reported to exhibit relevant antifungal properties, and many of them are classified as "Generally Recognized as Safe" (GRAS) by the U.S. Food and Drug Administration (FDA) ^45^.

Essential oils are not pure subtances so it is difficult to determine their mechanisms of action. However, their antimicrobial properties are related to their terpenic constituents since these lipophilic and low-molecular-weight compounds can alter the cell membrane and wall, thus inhibiting fungal sporulation and germination ^16^.

Accordingly, in this study, we evaluated the *in vitro* antifungal activity of the 27 constituent compounds of essential oils, including terpenoids and other structurally similar molecules. The results allowed us to recognize ten compounds with minimum inhibitory concentrations below 500 µg/ml for *A. alternata.* For *F. oxysporum,* only three compounds had inhibitory values under this range, indicating the higher sensitivity of the *Alternaria genus* to the terpenoids presence.

The results for both microorganisms quantitatively demonstrate that phenolic groups contribute to antifungal activity, with thymol and carvacrol being the most significant. These two compounds are abundantly present in oregano and thyme essential oils. For example, the efficacy of the essential oil derived from the Mexican oregano *(Lippia berlandieri* Schauer) has been evaluated against *Penicillium, Geotrichum, Aspergillus,* and *Rhizopus*^46^^,^^47^, and again, the phenolic compounds carvacrol and thymol are mainly responsible for the antifungal activity ^48^^,^^49^. Other studies showed that thyme essential oil and thymol display relevant antifungal activities against *A. alternata,* inhibiting mycelial growth ^50^ at minimum inhibitory concentrations below 200 µg/ml ^51^.

Other studies evidenced the *in vitro* antifungal activity of thymol on *Fusarium* spp. A recent study showed that thymol can inhibit the mycelial growth of *F. oxysporum,* finding a half-maximal inhibitory concentration (IC_50_) of 26.4 mg/L. This same study reported an 80% reduction of *F. oxysporum* germination induced by a thymol concentration of 60 mg/L ^52^. Another study found thymol-induced inhibition of conidia production and *Fusarium graminearum* hyphae growth at a mean effective concentration (EC_50_) of 26.3 µg/ml for 59 isolates of this microorganism ^53^.

As mentioned, the results obtained from the QSAR models showed the importance of the phenol group in antifungal activity. This finding was confirmed in the activity differences between thymol and carvacrol compared to other aromatic compounds. A relevant difference in activity was observed when compared to its precursor, p-cymene. It has a benzene group but lacks the hydroxyl to form a phenol.

The importance of the phenolic group can be appreciated when comparing the activity of thymol with menthol since both compounds have the hydroxyl group in the same position. However, menthol lacks the aromatic ring to form the phenol, resulting in decreased antifungal activity.

The hydroxyl group position in the phenol has relevant ramifications for biological activity: When it is in the meta position, it displays more significant antifungal activity than in the ortho position. This activity difference, with thymol having more antifungal activity than carvacrol, has also been reported against *Botrytis cinerea, Colletotrichum acutatum, Botryodiplodia theobromae,* and *Aspergillus niger*^54^^-^^56^.

Another structural characteristic related to the antifungal activity is observed when the hydroxyl groups (nOH) are replaced by ketone groups (nCO). The QSAR models showed that the ketone groups have an indirect relationship with biological activity, becoming evident when analyzing how the minimum inhibitory concentration and minimum fungicidal concentration values for geraniol, citronellol, and menthol increase in comparison with geranial, citronellal, and menthone. In particular, the five compounds with the most significant activity against *F. oxysporum* have phenolic and hydroxyl groups.

However, in the case of *A. alternata,* carvone is a rule exception since it contains a ketone group but is the fourth compound with the greatest antifungal activity. A possible explanation is the presence of conjugated non-aromatic carbons (nCconj), and their unsaturation index (Ui), considered by the QSAR models as relevant structural and topological features in antifungal activity. Although myrcene is the compound found to have the fifth highest activity level against *A. alternata,* it does not have a hydroxyl group. However, the carbon conjugation, its unsaturation index, and its lipophilicity values can be related to its biological activity.

Another relevant structural feature in biological activity considered by the QSAR models is the presence of the benzaldehyde group. It was observed how anisaldehyde and cuminaldehyde exhibited relevant activity against *A. alternata,* both with minimum inhibitory concentrations below 500 µg/ml. However, in the case of *F. oxysporum,* these compounds presented activities above 800 µg/ml.

In contrast, Ghose-Crippen octanol-water partition coefficient (AlogP) and topological descriptors, such as UNIP and Ui, contributed directly.

Regarding the molecular properties related to antifungal activity, those descriptors associated with the molecule's polarity, such as the Ghose-Crippen molar refractivity (AMR), the total absolute charge (Qtot), or the hydrophilic factor (HY) (such as AMR, Qtot, or Hy), were found to be only indirectly related. In contrast, the Ghose-Crippen octanol-water partition coefficient (AlogP) and topological descriptors, such as unipolarity (UNIP) and unsaturation index (UI), contributed directly. Molecular hydrophobicity, usually quantified as logP (the logarithm of the 1-octanol-water partition coefficient), is an important molecular characteristic in drug discovery. Ghose-Crippen octanol-water partition coefficient (AlogP) is one of the most widely used methods for estimating logP. This descriptor plays a significant role in the mechanism of action of essential oils.

The hypotheses suggest that the hydrophobic character of terpenes and their derivatives allows them to accumulate in the cellular lipid bilayer and generate changes in its permeability, causing the subsequent death of the microorganism ^57^. Likewise, its lipophilic character could alter the synthesis of ergosterol or a-glucans, inhibiting the formation of the cell membrane and wall (58). However, essential oils and their constituents have other effects, such as forming reactive oxygen species, inhibiting efflux pumps, and causing the dysfunction of fungal mitochondria ^59^^,^^60^. In the case of *F. oxysporum,* thymol induces the accumulation of superoxide radicals with a consequent increase in the activity of antioxidant enzymes and lipid peroxidation ^61^.

Despite the current evidence, we need more research to elucidate the cellular effects and potential biological targets behind the antifungal activity of essential oils and their constituents. Molecular modeling tools help to reduce the time and costs required to reveal the structural and molecular properties behind the antifungal activity and its mechanism of action. Therefore, it is proposed that future research continue the study of thymol and carvacrol using the tools of screening and molecular dynamics to identify possible biological targets and the generation of analogous compounds under the rational design. At the same time, more experimentation is required on these compounds' effects on *Alternaria* spp. since most of the found reports focus on *Fusarium* spp. or other *genera,* such as *Aspergillus* spp., *Penicillum* spp., or *Candida* spp.

The present study demonstrated the antifungal activity of terpenoid compounds against *A. alternata* and *F. oxysporum,* contributing to the identification of the structural characteristics required for these compounds to exert their antifungal action. The obtained experimental evidence and the proposed mathematical models provide promising tools for developing new antifungal agents, mainly derivatives of phenolic terpenoids, such as thymol and carvacrol. However, it remains necessary to continue experimental and computational studies to optimize the potential of such compounds and elucidate their mechanisms of action.
